# Immune dysregulation in *Mycoplasma pneumoniae* pneumonia: mechanistic controversies and clinical translation from inflammatory dysregulation and immune evasion to chronic injury

**DOI:** 10.3389/fimmu.2026.1724496

**Published:** 2026-02-25

**Authors:** Xuejun Li, Yudong Wang, Qiuyan Wang, Hongji Wu, Yongbin Yan, Yibai Xiong, Ying Ding

**Affiliations:** 1 Pediatric Hospital, The First Affiliated Hospital of Henan University of Chinese Medicine, Zhengzhou, China; 2 Pediatric Medical College, Henan University of Chinese Medicine, Zhengzhou, China; 3 Institute of Chinese Materia Medica, China Academy of Chinese Medical Sciences, Beijing, China

**Keywords:** Mycoplasma pneumoniae, immune dysregulation, immune evasison, excessive inflammation, chronic injury, clinical translation

## Abstract

*Mycoplasma pneumoniae* (MP) is a leading cause of pediatric community-acquired pneumonia, with clinical manifestations ranging from self-limiting disease to severe refractory pneumonia and long-term pulmonary sequelae. Three interrelated, partially overlapping yet still contested processes can explain the core pathogenic mechanisms of MP pneumonia (MPP). In the acute phase, immune dysregulation is characterized by excessive cytokine release and abnormal activation of innate and adaptive immune cells; however, the origin and regulation of this excessive inflammation remain controversial. During the immune evasion phase, MP employs multiple escape strategies, including adhesion proteins, CARDS toxins, and genomic plasticity, to circumvent host defenses, establish persistent infections, and further leave hidden dangers for acute phase inflammatory dysregulation and chronic phase structural remodeling. However, the exact molecular mediators remain unclear. Macrolide antibiotics remain the primary clinical treatment; however, therapeutic limitations persist owing to increasing drug resistance and the lack of immunopathological interventions. In the migration phase, sustained immune activation and abnormal repair processes persist even after pathogen clearance, resulting in chronic lung injury and fibrosis, with underlying immunological mechanisms still poorly understood. This review synthesizes current insights into immune dysregulation across the acute-to-chronic spectrum of MPP, identifies unresolved immunopathological bottlenecks, and highlights translational opportunities for immune-targeted interventions beyond antibiotics.

## 1 Introduction

*Mycoplasma pneumoniae* (MP) is a major pathogen causing community-acquired pneumonia (CAP) worldwide, accounting for up to 40% of CAP cases in children aged five years and older (1). In China, the incidence of MP-associated CAP in children ranges from 15% to 37%, with the majority occurring in the 5–10 age group (2). Notably, the incidence of MP pneumonia (MPP) has markedly increased since the COVID-19 outbreak (3, 4). Although MPP is often self-limiting, its clinical manifestations are complex and heterogeneous. Severe cases may rapidly progress to refractory MPP (RMPP). Approximately 25% of MP-infected individuals experience extrapulmonary complications at various stages due to infection spread or autoimmune mechanisms, including hemolytic anemia, acute demyelinating encephalomyelitis, Stevens-Johnson syndrome, and septic arthritis (5, 6). Pathological damage caused by host immune dysregulation or “excessive inflammation” is considered a core driver of MP pathogenicity (6). In particular, MPP tends to be prevalent among children, causing more severe clinical symptoms and inflammatory pathological damage, with higher risk of extrapulmonary complications and macrolide antibiotic resistance, suggesting age-related immunological differences (7, 8).

Emerging clinical and experimental studies indicate that MPP pathogenesis is rooted not in direct damage by pathogens but in immune homeostasis imbalance (9). This immune imbalance persists throughout the disease course, manifesting in three interrelated yet distinct pathological phases: inflammatory dysregulation or excessive inflammatory response during the acute phase; persistent infection due to immune evasion; and chronic immune injury with abnormal tissue repair during the convalescence or recovery phase (10, 11). However, the three pathological states are not absolutely continuous over time; they may partially overlap, coexist, or cycle, with unclear boundaries, forming a heterogeneous clinical spectrum ranging from acute pneumonia to extrapulmonary complications and long-term sequelae (6). The dynamic equilibrium of multiple factors—including host immune response, pathogen load, and site of colonization—is pivotal in determining the specific pathological phase of MPP (6). Although this “immune imbalance” framework provides a critical perspective on the complex pathophysiology of MPP, significant mechanistic controversies and clinical translation challenges remain regarding its specific mechanisms, dynamic transition conditions between pathological stages (immunological thresholds, clinical markers, or changes in tissue levels, etc.), and the impact of individual variation (host heterogeneity) on outcomes.

**Figure 1:**
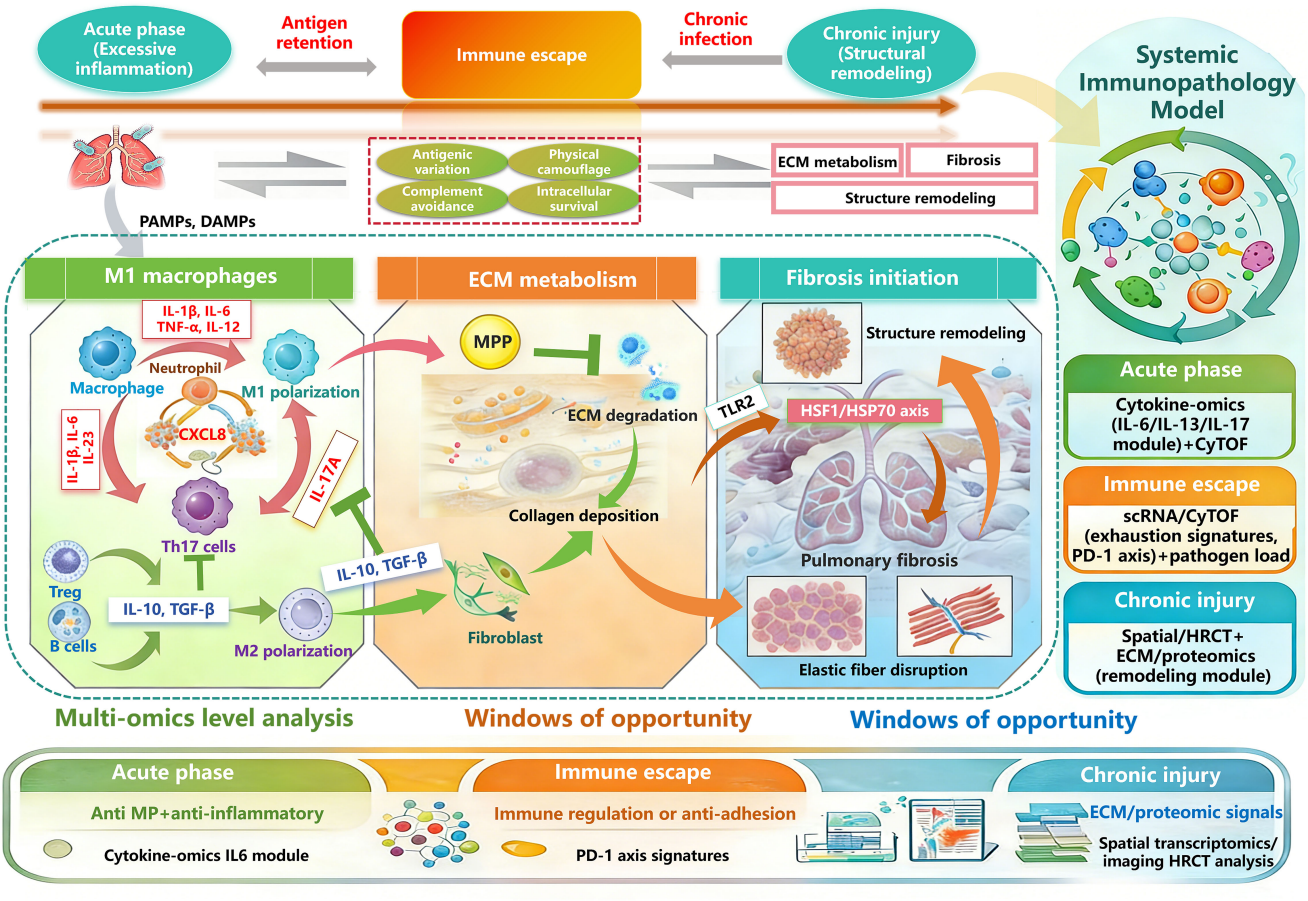
System orientation diagram.

This review systematically synthesizing recent advances and key academic controversies regarding the three dynamically sequential, partially overlapping pathological stages in MPP (i.e., “immune evasion-inflammatory dysregulation-chronic injury”). It describes key molecular events and cellular mechanisms, explores potential targeted intervention strategies, discusses possible conditions for dynamic transitions between the three pathological stages, and evaluates their translational potential and associated challenges. The aim is to provide a theoretical basis for testable systems models of different pathological stages of MPP, thereby offering new perspectives for future research and clinical practice in this field. We provide a systematic orientation diagram for this review (**Figure 1**).

## 2 Pathogenic factors of MP

### 2.1 Adhesion proteins

**Figure 2:**
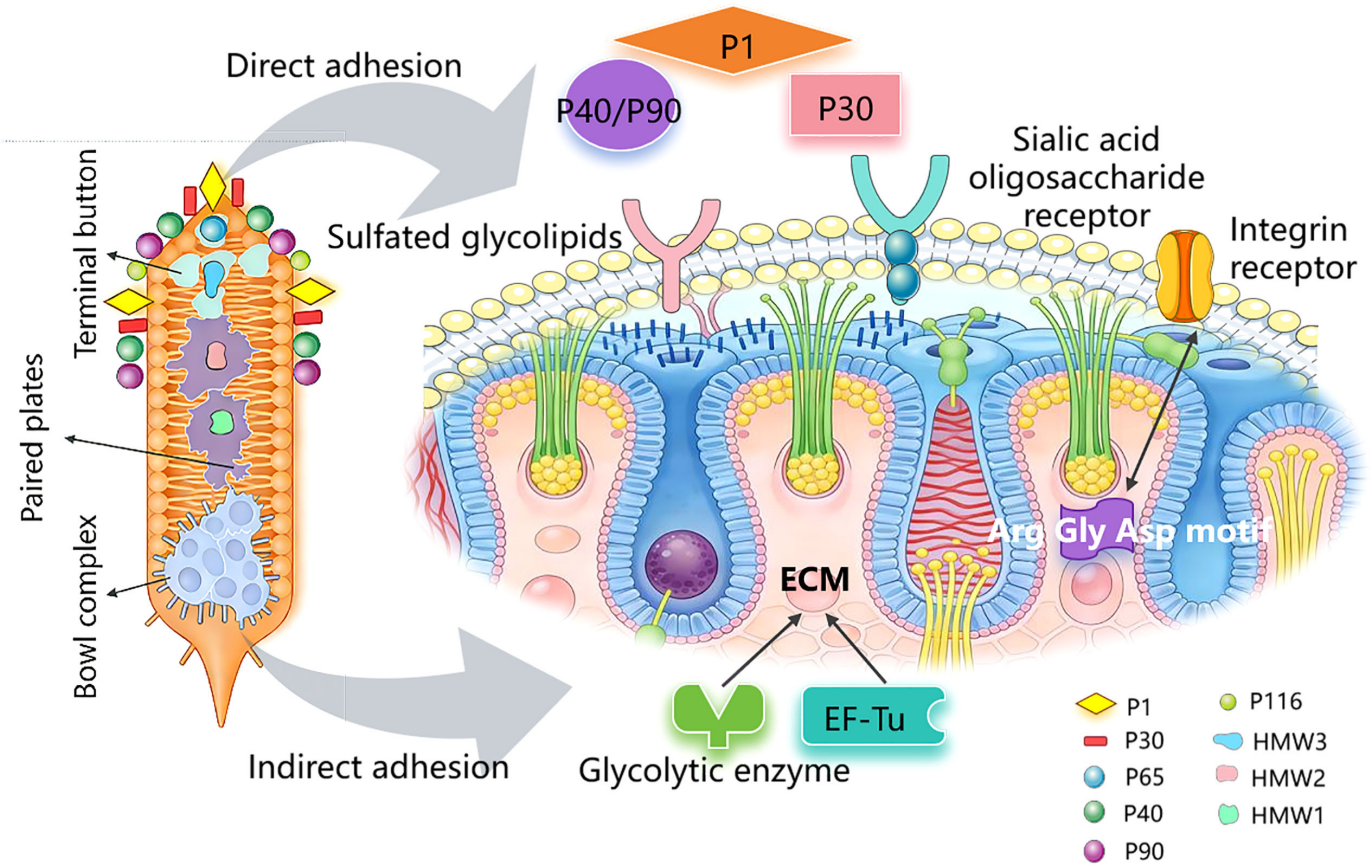
MP adhesion protein mediates adhesion by binding to host cell surface receptors.

The successful colonization of MP in the host respiratory tract depends on the bacterial tip’s adhesion organelles and the highly specialized adhesion-related proteins on its surface. The adhesion organelles comprise internal and surface structures (**Figure 2**). The surface structures mediate attachment to host cell surfaces and include P1, P30, and P40/P90, which together form the Nap structure within the adhesion complex. P1, P30, P40/P90 are the main adhesins responsible for recognizing sialic acid-containing oligosaccharide receptors and sulfated glycolipids on host cell surfaces (12). Their primary roles include reinforcing adhesion and triggering intracellular metabolic shifts and ultrastructural modifications in infected cells (13). P1 can also bind to vimentin on host respiratory epithelial cells, promoting adhesion and migration (14). The internal structures comprise a translucent zone and a core structure. The core structure consists of the terminal button (HMW3 and P65), which determines the direction of MP sliding; the paired plates (HMW1, HMW2, CpsG, and HMW3), which form the attachment scaffold; the bowl/wheel complex (P24, P41, P200, TopJ, Lon, and MPN387), which generates and transmits force.

#### 2.1.1 P1

P1 serves as the primary pathogenic factor of MP. Its N-terminal extracellular domain features a seven-leaf β-helical fan structure and mainly facilitates host receptor recognition and binding. The central repeat sequence is involved in protein–protein interactions, and the C-terminal domain includes transmembrane regions and a cytoplasmic anchor that anchors the protein and confers strong immunogenicity (12). Upon MP contact with target cells, the P1 precursor is rapidly cleaved into its mature form, which binds to the host cell’s sialic acid oligosaccharide receptors to mediate adhesion. Concurrently, it promotes the release of CARDS Tx, hydrogen peroxide (H_2_O_2_), and superoxide anion (O_2_^−^), causing damage to host cells (11). HEp-2 cells are commonly used in MP adhesion studies and require surface exposure of N- and C-terminal regions of P1 (15, 16). The adhesion epitope resides at the C-terminus of P1, and mRNA vaccines targeting this region (amino acids 1288–1518) have emerged as promising candidates for combating MPP (17).

#### 2.1.2 P30

P30 comprises four regions: a propeptide region, an intracellular segment (Domain I), the transmembrane region, and an extracellular region (Domains II and III). Domain I, located at the N-terminus, functions as a surface-binding component that connects the organelle core to the P30 C-terminus; its deletion results in loss of sliding motility and adhesion (18). P30 forms an adhesion complex with P1 and undergoes conformational folding to mediate adhesion to host cells (19). The extracellular domains at the N-terminus of P30 can bind to salivary acidified oligosaccharides and sulfated oligosaccharide receptors (20), enhancing adhesion strength. P30 interacts closely with P1 and is essential for maintaining the structural integrity and functionality of the tip-adhesion complex.

#### 2.1.3 P116

P116 adopts a unique fold. Its semi-open left-handed core domain comprises four pairs of antiparallel amphipathic α-helices. The N-terminus forms a “thumb” domain containing RepMP4/5 repeats that mediate adhesion, while the dimer interface constitutes a “wrist” domain that interacts tightly with adjacent monomers (21). P116 exhibits strong immunogenicity and functions independently of P1 and P30. Its C-terminal fragment is commonly used in serological diagnosis, while the 27 kDa N-terminal fragment represents one of the immunodominant regions. This fragment holds promise for MP serodiagnosis and is considered a leading antigen candidate for vaccine design (22).

#### 2.1.4 P40/P90

P40 and P90 proteins are proteolytic derivatives of the common precursor protein, Mpn142. They share similar structural architectures, comprising a large extracellular domain (crown), transmembrane helices, and a short cytoplasmic tail (C-domain). The extracellular region forms the N-terminal segment of a heptameric β-helical crown structure, which harbors a sialic acid-binding site (23). Additionally, P40 and P90 function as immunodominant proteins during human MP infection. Genetic variations within their terminal surface-exposed domains contribute to clinical symptom variation. Both proteins exhibit strong reactivity to human infection sera, providing new strategies for vaccine development against MP infection (12).

#### 2.1.5 P65

Region III of P65 is the C-terminal domain, forming a structure containing an α-helix (24). This domain interacts with P30 to maintain its stability, provides mechanical support for the attachment organelle, and ensures P1 enrichment at the cell apex. Region I is an N-terminal intrinsically disordered region containing an acidic proline-rich (APR) domain that confers a unique rigid extension structure to the P65 protein (25). In SDS-PAGE, this rigid structure impedes complete SDS binding and protein coiling, increasing the hydrodynamic radius and resulting in a significantly lower migration rate than globular proteins of the same molecular weight (25). In biological function, this rigid extension may function as a molecular “scaffold” or “arm, “ fully exposing and extending the C-terminal RGD motif beyond the bacterial surface. This effectively overcomes steric hindrance, facilitating specific binding to host integrins and assembly of the gliding complex (25). Thus, although “slow migration” is an *in vitro* phenomenon, it reveals the mechanism by which P65 efficiently performs binding and gliding functions by reflecting key structural features of the APR. Truncation of P65 reduces MP sliding velocity and disrupts terminal organelle protein homeostasis (26). The M129 strain, which encodes the P65 amino acid sequence, activates the release of proinflammatory cytokines in host cells, thereby contributing to tissue damage (27). Although P65 contains partially surface-exposed regions that are immunogenic and antigenic, its suitability as a vaccine target or diagnostic marker remains controversial.

#### 2.1.6 High molecular weight proteins 1–3

High molecular weight proteins 1–3 (HMW1–3) collectively form the internal scaffold structure of the adhesion organelle. HMW1 central domain features an APR motif that facilitates irregular migration (28, 29). HMW1 indirectly contributes to adhesion by regulating P1 localization within the attachment organelles. It is also phosphorylated by Ser/Thr kinase in an ATP-dependent manner to promote sliding movement (30) and influences the stability of P65 protein levels (25). The N- and C-termini of HMW2 form characteristic α-helix-in-helix structures that mediate protein oligomerization, support the mechanical stability of adhesion organelles (31), and transmit contractile forces from the bowl-shaped complex to P1 and P30, thereby enhancing host cell binding and adhesion. Loss of HMW2 leads to reduced stability and accelerated turnover of proteins such as HMW1, HMW3, P65, and P30 (29, 32). HMW3 primarily consists of β-folds and ring/turn structures (33). By maintaining the structural integrity of adhesion organelles and forming complexes with the P65 protein, HMW3 facilitates MP attachment to respiratory epithelial cells and protects against ciliary clearance. Its absence leads to reduced P65 levels and dispersed localization, which hinder the ordered aggregation of P1 at terminal organelles and reduce adhesion (34), thereby offering a novel approach for anti-adhesion therapy.

**Table 1** presents relevant information and research applications of MP adhesion proteins.

**Table 1:** MP-related adhesion proteins.

Adhesion-related protein	Character	Positioning	Molecular weight (KD)	Secondary structure	Biological function	Pathogenic mechanism	Research applications
P1	Cell membrane major adhesion protein	Adhesion organelle tip	170	Transmembrane β-barrel domain, C-terminal α-helix	Binds to host sialic acid receptors, mediating adhesion and sliding	Initial adhesion, immune attack	Serological testing, vaccine core antigen, drug target screening, adhesion mechanism research model, strain typing basis.
P30	Organelle tip proteins	Adhesion organelle tip	30	N-terminal signal peptide, central α-helix bundle	Stabilizes P1, maintaining the integrity of the adhesion complex; mediates sliding.	Direct adhesion, immune evasion	Pathogenicity assessment markers, research on the assembly mechanism of the adhesion complex, attenuated vaccine candidate components, and studies on pathogenicity-related genes.
P116	Organelle bottom anchoring protein	Adhesion organelle tip	116	Extracellular domain rich in β-folds	Stable adhesion device	Enhance adhesion efficiency, immune evasion, and inflammation induction	Gene knockout model studies, adhesion mechanisms, immunoblotting diagnostic antigens, and exploration of cytotoxic mechanisms
P40/P90	Transmembrane protein complex	Adhesion organelle tip	40/90	P40: Transmembrane α-helix; P90: Globular structure	Drive sliding, form transmembrane composite, stabilize P1 function	Adhesion maintenance, receptor recognition, immune evasion	Stability study of adhesive complexes, sliding mechanism study, screening of anti sports drug targets
P65	Surface-variable protein	Terminal button	65	N-terminal APR, C-terminal α-helical domain	Stabilize P30, maintain cell polarity and motor coordination.	Adhesive support, pro-inflammatory effect, immune escape	Epidemiological tracking, research on immune evasion mechanisms, and exploration of exercise mechanisms
HMW1	Cytoskeletal-associated proteins	Thin sheets of paired plates	180–220	Curled spiral structural domain, central APR	Stabilize the cytoskeleton and regulate the distribution of adhesion proteins	Indirect adhesion, P1 recruitment	Exploration of Gene function research model and toxicity factor regulation mechanism
HMW2	Cytoskeletal proteins	Thick plates of paired plates	210	Extended α-helix bundle	Maintain the integrity of cell morphology and transmit adhesive contraction force	Adhesion enhancement, immune escape, pro-inflammatory response	Research on cellular morphogenesis and antimicrobial susceptibility targets
HMW3	Cellular organelle base protein	Terminal button	190	APR, β folding and circular/angular	Maintain the structural integrity, sliding motion, and cell division of adherent organelles	Indirect adhesion, long-term colonization	Structural biology research, engineering strain construction

### 2.2 Community-acquired respiratory distress syndrome toxin

CARDS Tx is composed of 17 α-helices and 43 β-strands organized into three isosceles triangular domains. Its N-terminal mART domain connects via a hinge and extensive interface to two tandem C-terminal β-trident subdomains (D2 + D3) (35). Upon binding to the surfactant protein A (SP-A) receptor on host cells, CARDS Tx undergoes rapid internalization via the clathrin-mediated pathway. Subsequently, it is transported retrogradely from the endosome through the Golgi complex to the endoplasmic reticulum, where it induces vesicle formation (36). Through ADP-ribosylation and vacuolation, CARDS Tx stimulates excessive immune-inflammatory responses following MP infection, leading to fibrillar stasis, cytoplasmic swelling and vacuolation, nuclear fragmentation, extensive inflammation, and histopathological damage (37). CARDS Tx promotes the release of proinflammatory mediators, including interleukin-1 beta (IL-1β) and IL-18, by activating NLRP3 inflammasomes, thereby enhancing Th1- and Th2-mediated inflammatory responses (37–40). It also activates the JAK/STAT1 signaling pathway, inducing M1 macrophages to secrete CXCL9, which recruits Th1 cells, forming a positive feedback loop that exacerbates airway hyperresponsiveness and lung injury (38). CARDS Tx can induce neutrophil infiltration through Toll-like receptor 2 (TLR2)-independent and IL-1α-dependent mechanisms (41). Its C-terminal region exhibits high serological discriminatory power and induces antibody responses during infection, highlighting its potential for diagnostic development, novel therapeutic targeting, and attenuated vaccine design (38, 42). CARDS Tx is increasingly recognized as a potential biomarker for assessing disease severity and prognosis.

### 2.3 Lipid-associated membrane proteins

LAMPs serve as a key interface for pathogen–host interactions and function as potent proinflammatory molecules. Classical membrane lipoproteins of LAMPs contain N-terminal diacylglycerocysteine motifs that anchor them to the cell membrane and allow recognition as ligands by TLR, thereby activating NF-κB and MAPK pathways (38). This leads to the release of proinflammatory cytokines or activates NLRP3 inflammasomes to induce IL-1β expression through a mechanism independent of Gasdermin D and pyroptosis (43). Hu et al. (44) reported that LAMPs stimulate Nrf2-silenced THP-1 cells to produce reactive oxygen species (ROS), nitric oxide (NO), IL-6, and IL-8, and upregulate heme oxygenase-1 expression, which induces Nrf2 nuclear translocation and inhibits the expression of inflammatory reactants in THP-1 cells. In vaccine research, Mara et al. (45, 46) identified the lipid moiety of lipoproteins as the cause of vaccine-enhanced disease in experimental MP vaccines. Mice immunized with LAMP vaccines exhibited elevated levels of TNF-α, IL-1β, IL-6, IL-17A, and the human IL-8 homolog, KC, in their lung lavage fluid, which led to IL-17A-dependent neutrophil recruitment and subsequent suppurative pneumonia. Exploring the synergistic pathogenic mechanisms of LAMPs and overcoming bottlenecks in vaccine design will provide targeted interventions and combined prevention strategies for MP infections.

**Figure 3:**
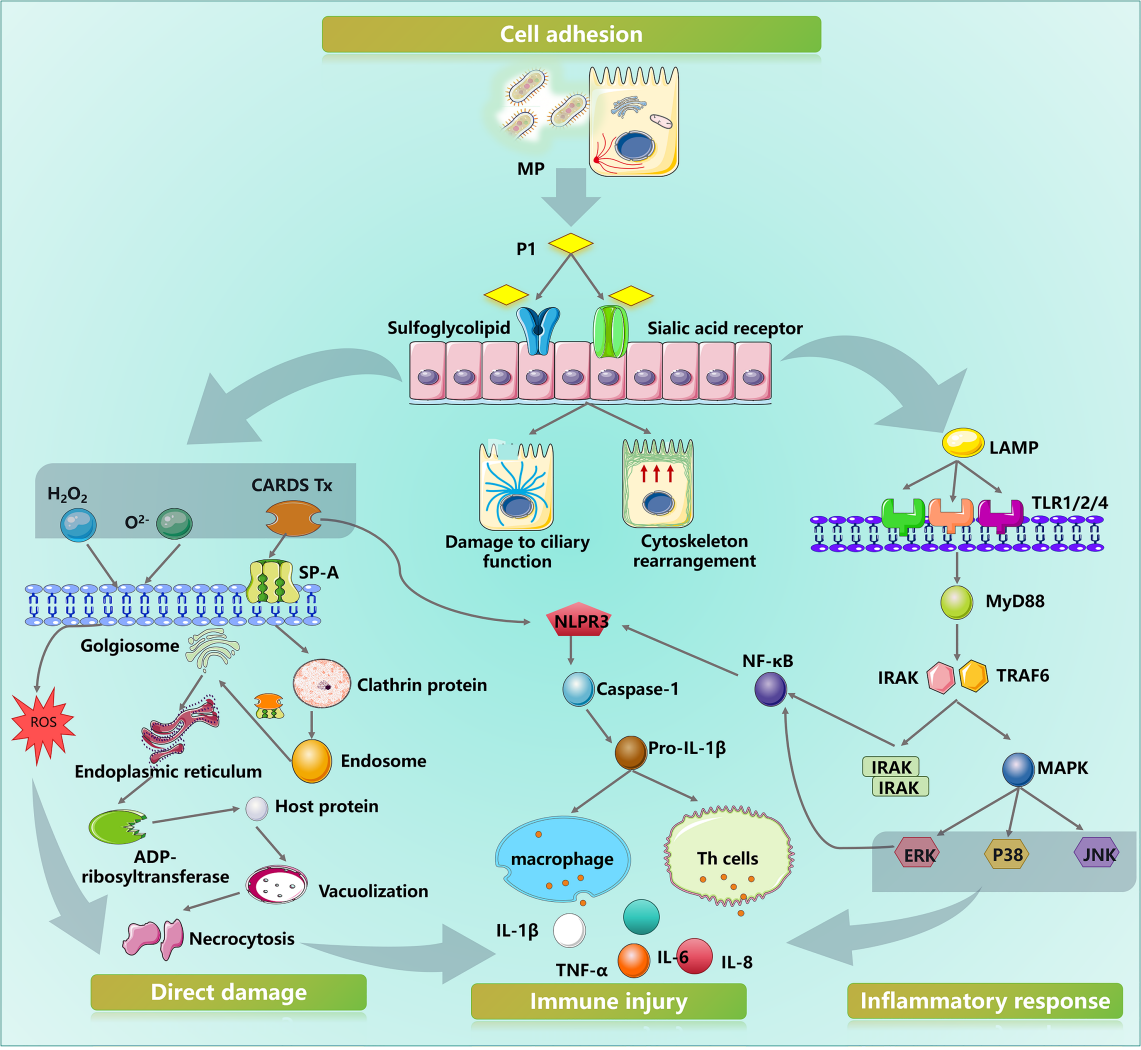
Schematic diagram of MPP-induced pulmonary immune injury.

In summary, despite its structural simplicity, MP triggers complex immunopathological responses during pulmonary infection through the synergistic action of multiple pathogenic factors (**Figure 3**).

## 3 Core mechanisms and controversial issues in MPP immune dysregulation

The systemic immune framework of MPP comprises acute dysregulation, immune evasion, and chronic injury processes with unclear temporal boundaries and partial overlap. However, unlike adults, children exhibit a unique age-related immune profile, demonstrating differences in structural, immune, and reparative capacities. This characteristic profoundly influences and exacerbates the aforementioned three pathological processes. First, in terms of airway structure, pediatric airways are narrower, exhibit disproportionate growth relative to lung parenchyma, possess fewer alveoli, and demonstrate weaker mucus clearance function, all of which facilitate pathogen colonization (47). Imaging studies more frequently reveal air bronchography, bronchial thickening, and atelectasis (48). At the immune level, in early childhood, innate immune responses serve as the core defense against infection. Their excessive activation generates inflammatory reactions, with inflammatory markers such as hsCRP and GlycA positively correlated with innate immune cell activity, leading to more intense early release of inflammatory cytokines (49). Concurrently, children exhibit immature adaptive immune development, making them more susceptible to Th2 response skewing (elevated IL-4/IFN-γ ratio) in CD4+ T cell responses, Tregs function remains relatively underdeveloped, and B cells and follicular helper T cells demonstrate delayed or inadequate antibody production, thereby weakening immune clearance efficiency and promoting immunopathological damage (50). Finally, during the recovery phase following injury, children’s tissue repair and immune regulation capabilities remain underdeveloped, making them more prone to prolonged recovery or residual sequelae. This comprehensive immunological disparity renders children more susceptible to excessive inflammation, subsequent immune exhaustion following MP infection, more severe clinical symptoms, broader intrapulmonary and extrapulmonary damage, and a higher incidence of the 23S rRNA A2063G mutation (51). Therefore, the age-related immunological background is a key foundation for understanding the immune imbalance in pediatric MPP.

### 3.1 Mechanisms of immune evasion in MP—the starting point of immune dysregulation

MP immune evasion can coexist with other pathological stages, lacking clear temporal boundaries. Determining MP immune escape requires comprehensive multidimensional criteria: (i) At the immunological threshold level, the core feature is effector T cell functional exhaustion. Single-cell RNA-seq and high-dimensional flow cytometry reveal depletion of KLRG1-high CD8+ T cell compartments and markedly increased granzyme expression in Th17 cells in MPP children, potentially explaining MP uncontrolled replication and severe pulmonary inflammation—i.e., immune evasion (52). (ii) Clinical evidence reveals that MP is difficult to clear and persists long-term (at least 4 months), particularly in the lower respiratory tract (53). This leads to prolonged disease course and poor response to macrolide antibiotics, correlating with airway injury and chronic sequelae. Persistently elevated IL-10/IFN-γ ratios suggest a shift in the immune response toward a direction unfavorable for pathogen clearance (54). (iii) At the tissue level, pulmonary histopathology may demonstrate a transition from an acute inflammatory infiltrate dominated by neutrophils to a chronic inflammatory infiltrate dominated by lymphocytes (55). In summary, immune evasion is not a singular event but a dynamic outcome resulting from the combined crossing of critical thresholds by pathogen load, host cellular immune dysfunction, and the local suppressive microenvironment. In particular, it is crucial for sustaining persistent infection during the chronic pathological damage phase downstream. MP has developed multiple strategies to evade immune surveillance, as detailed below.

#### 3.1.1 Antigenic variation as a strategy to evade humoral immune surveillance

One of the immune-evasion strategies employed by MP involves its unique components and virulence factors, which achieve antigenic variation through structural alterations or immune interference (56). P1 protein-encoding gene possesses highly variable regions and abundant repetitive sequences that undergo frequent mutations, making it the primary driver of antigenic variation (57). The *p1* operon contains *p1* and *orf6* genes, which encode P1 and P40/P90 proteins, respectively. Both genes contain repeat element regions (RepMP4, RepMP2/3, and RepMP5) that undergo homologous recombination to facilitate immune escape (58). RecAMp and RecAMg enable recombination between repetitive DNA elements, leading to antigenic surface protein variation and subsequent evasion of the host’s humoral immune response (59). CARDS Tx can trigger autoimmune reactions through molecular mimicry (60), thereby overwhelming the immune system with severe toxin-induced inflammation and autoimmune responses. This reduces recognition pressure on MP self-antigens, providing an evolutionary time window and selective advantage for P1 antigen variation. LAMPs rely on their structural domain variations to offer space for antigenic variation. Using lipoprotein lipase (an exogenous water-soluble enzyme) to remove the acyl chain from MP lipoproteins, thereby decoupling it from the protein domain, reduces NF-κB activation by approximately 60% (61), though its *in vivo* relevance remains uncertain. The lipopeptide MPPL-1, synthesized by Into et al. (62)—composed of S-dipalmitoylglycerylcysteine residues conjugated to diverse peptide sequences—contains conserved sequences characteristic of the MP-specific homologous lipoprotein family; however, its cytokine-inducing activity is extremely weak and does not antagonize TLR2 recognition of the lipopeptide FSL-1. This lipid domain can influence the inflammatory cascade and antigen-presenting efficiency, enabling MP to evade host immune surveillance.

#### 3.1.2 Active immune recognition interferes with innate immune responses

MP can synthesize capsular polysaccharides (CPS) similar to those on host cell surfaces, forming a physical barrier that conceals its highly immunogenic LAMPs and lipopeptides from TLR recognition (63). Additionally, it can exploit the DC-SIGN signaling pathway to evade immune surveillance (63). However, this mechanism remains controversial, as MP’s CPS synthesis varies among strains, and evidence for its specific chemical composition and immunomodulatory functions is derived from indirect experiments. For example, CPS extracted from M129 has been shown *in vitro* to induce IL-10 secretion and inhibit dendritic cell maturation via the DC-SIGN pathway (64), but this cannot be fully equated with the role of GPS on the surface of natural bacteria within the infectious microenvironment. Secondly, research on MP CPS remains scarce, with most evidence stemming from serological studies of other MP components: specific epitopes of P1 adhesin may trigger cross-immunological reactions by mimicking host metabolic enzymes such as glyceraldehyde-3-phosphate dehydrogenase (65); The myelin lipid galactocerebroside (Gal-C)-like glycolipid structure present in MP may also cause its specific antibodies to cross-react with host neural tissues (66). Furthermore, the C-terminal regions of P1 and P30 proteins exhibit high homology with host myosin, cytoskeletal proteins, keratin, and fibrinogen. By mimicking these self-antigens, MP evades direct immune system attacks, thereby impairing pathogen clearance and promoting persistent infection (38). MP also evades complement-mediated killing. MP actively recruits factor H to its surface, accelerating the dissociation of the C3/C5 convertase and serving as a cofactor for complement factor I-mediated C3b degradation, thereby suppressing the amplification loop of complement activation and allowing MP to escape immune attack (67). Moreover, oxidative stress is dual in this immune response: on the one hand, MP synthesizes and releases large amounts of H_2_O_2_, directly damaging host cells; on the other hand, MP has evolved a sophisticated antioxidant enzyme system. This system includes superoxide dismutase (SOD) and catalase-like proteins—SOD converts superoxide anion (O_2_^−^) into H_2_O_2_, while catalase-like proteins decompose excess H_2_O_2_ (68). Through this synergistic “generation and clearance” mechanism, MP not only utilizes H_2_O_2_ for attack but also effectively neutralizes the critical toxicity of ROS to itself, thereby sustaining survival under host immune assault.

#### 3.1.3 Intracellular survival provides a key pathway for immune evasion

In addition to sialic acid oligosaccharide receptors, MP also expresses multiple surface-exposed glycolytic enzymes, including lactate dehydrogenase, phosphoglycerate mutase, pyruvate kinase, and glyceraldehyde-3-phosphate dehydrogenase, which can interact with extracellular matrix (ECM) components to promote adhesion processes (69, 70). The extension factor Tu (EF-Tu) on the MP surface can also bind to fibronectin through its carboxyl-terminal region, mediating interactions between the bacterium and the extracellular matrix (71). The integrin transmembrane receptor family, widely expressed in host cells, recognizes the Arg-Gly-Asp motif in the ECM and mediates cell anchoring through bidirectional mechanotransduction (69). The aforementioned tight adhesion mechanism can actively trigger the host cell’s endocytic function, causing MP to be “actively” drawn into the cell. CARDS Tx, when combined with SP-A, enables MP to penetrate the host barrier and permanently attach to target cells such as alveolar macrophages and type II alveolar epithelial cells (72). This allows manipulation of the host cell’s actin cytoskeletal rearrangement, promoting the internalization process, and ultimately leading to its settlement within membrane-bound intracellular vesicles. The dual action of apoptosis and autophagy also creates opportunities for MP survival. MP can activate p38 MAPK/mitochondrial apoptosis to promote pulmonary epithelial cell apoptosis through CRP overexpression (73); however, it can also inhibit host cell apoptosis by influencing Bcl-2/Bax expression via the mitochondrial apoptosis pathway (73, 74). *Mycoplasma bovis* can interfere with the unfolded protein response signaling pathway through the glycine cleavage system H protein, this suppresses the expression of the pro-apoptotic molecule CHOP and blocks endoplasmic reticulum-mediated intrinsic apoptosis (75). In terms of autophagy regulation, although MP activates autophagosome formation by increasing LC3-II conversion, Beclin-1 expression, and autophagosome numbers, it blocks autophagic flux through unknown mechanisms, preventing autophagosome–lysosome fusion and leading to the accumulation of the autophagic substrate p62 (76). MP can establish resilient shelters within various cellular locations—including vesicles, the cytoplasm, perinuclear regions, and even the nucleus—thereby shielding themselves from circulating antibodies, complement components, and specific antimicrobial peptides. This reduces their susceptibility to clearance by extracellular immune mechanisms (77).

Although the precise mechanisms underlying MP intracellular survival remain incompletely understood, it employs multiple sophisticated strategies to facilitate its survival. Beyond adhesion, internalization, incomplete autophagy, and intracellular localization strategies (36, 78), MP also utilizes intracellular permeation to penetrate host cell membranes and acquire nutrients to sustain its survival (10). MP has also evolved the MPN400 immunoglobulin-binding protein (IbpM) to bind host IgG, IgA, and IgM for immune evasion (79) or employs Mpn491 to evade neutrophil killing (80).

The intracellular lifestyle of MP leads to profound immune evasion effects, centered on alterations in antigen presentation pathways—particularly restricted MHC class I presentation and the absence of CD8+ T cell responses. MHC-I-associated peptides primarily originate from ubiquitinated proteins degraded via the proteasome-dependent pathway (81). These serve as cytoplasmic antigens, whose cross-presentation is primarily mediated by specific DC subsets through adaptation of their endocytic and phagocytic pathways to initiate CD8+ T cell responses (82). MP is encapsulated within vesicles, making its antigens less susceptible to cytoplasmic processing by proteasomes, and MP antigenic peptides are primarily generated within phagosomes/endosomes and tend to be presented to CD4+ T cells via the MHC class II pathway (83). Lung function and histopathological changes mediated by CARDS Tx also depend on CD4+ T cells (60). Therefore, MP peptides are difficult to present to CD8+ T cells via MHC class I molecules. This deficiency in MHC class I cross-presentation results in a weakened or inadequate CD8+ cytotoxic T cell response specific to MP, preventing effective clearance of infected host cells. It also provides MP with an opportunity to achieve immune escape and sustain infection, laying the groundwork for subsequent chronic infection and structural remodeling.

#### 3.1.4 Active suppression of adaptive immunity constructs a tolerant microenvironment

MP infection induces immunosuppression or immune tolerance by modulating the host’s adaptive immune response. MP regulates the expression of the endogenous stress factor HMGB-1 through binding to SP-A (84) or induces DC maturation via TLR2/MyD88/NF-κB signaling through its lipoprotein components (85, 86), thereby increasing the number of activated T and B cells and mediating excessive inflammatory responses. However, CPS can bind to DC-SIGN as ligands, thereby inhibiting DC maturation and enhancing their phagocytic activity. This results in the downregulation of co-stimulatory molecules such as CD80, CD86, and CD83 and alterations in cytokine secretion profiles, ultimately reducing the capacity of DCs to activate naive T cells (87). Dysfunctional DCs fail to initiate T cell responses effectively and may also promote the differentiation of Tregs, indirectly inducing immune tolerance (88).

MP infection results in T cell functional suppression and imbalance in subset proportions. Studies have revealed reduced CD3^+^ and CD4^+^ T cell populations and elevated CD8^+^ T cells in bronchoalveolar lavage fluid (BALF) from patients with MPP (89). Despite excessive T-cell activation during the acute phase of MPP, disease progression ultimately leads to T-cell apoptosis and exhaustion (90). Study reported a reduction in the CD4+/CD8+ ratio in children with plastic bronchitis (PB), a rare and severe complication of MPP (91). MP infection also induces Th-2-type allergic inflammation. Recombinant CARDS Tx induces expression of Th-2 cytokines IL-4 and IL-13, as well as Th-2 chemokines CCL17 and CCL22, thereby triggering a mixed cellular inflammatory response characterized by the accumulation of eosinophils, T cells, and B cells (60). This Th2 shift can weaken the Th1 response and hinder pathogen clearance (92). MP can nonspecifically stimulate B cells to produce polyclonal immunoglobulins, including IgM, IgA, and IgE (93). More importantly, since MP can cross-react with the host and generate autoantibodies, the latter bind to corresponding antigens to form circulating immune complexes that trigger Type III hypersensitivity reactions. This further activates the complement system and causes inflammatory damage, thereby misdirecting the immune attack from pathogens to the host’s own tissues, representing a key mechanism by which MP induces various extrapulmonary complications (94).

Although MP has developed multiple immune evasion strategies, host-related age-associated immune differences also contribute to this pathological process to some extent (95). The immune systems of juvenile children are in a developmental and training phase (96), with alveolar macrophages exhibiting lower phagocytic clearance efficiency, limited antigen experience, and delayed adaptive immune activation (97). This age-determined state of “immunological immaturity” has led to the hypothesis that it might create a permissive environment for early MP immune evasion. This hypothesis, if substantiated, could inform future translational medicine research, suggesting that age-dependent immune stratification might be explored as a potential strategy to refine management approaches for MPP. A direct causal link between pediatric immune maturation status and *in vivo* MP escape remains to be empirically established. Furthermore, before any immune-modulatory strategy derived from this concept could be considered, its long-term safety in the context of the developing pediatric immune system must be rigorously evaluated through comprehensive preclinical and early-phase clinical studies.

#### 3.1.5 Controversial issues targeting immune evasion

**Figure 4:**
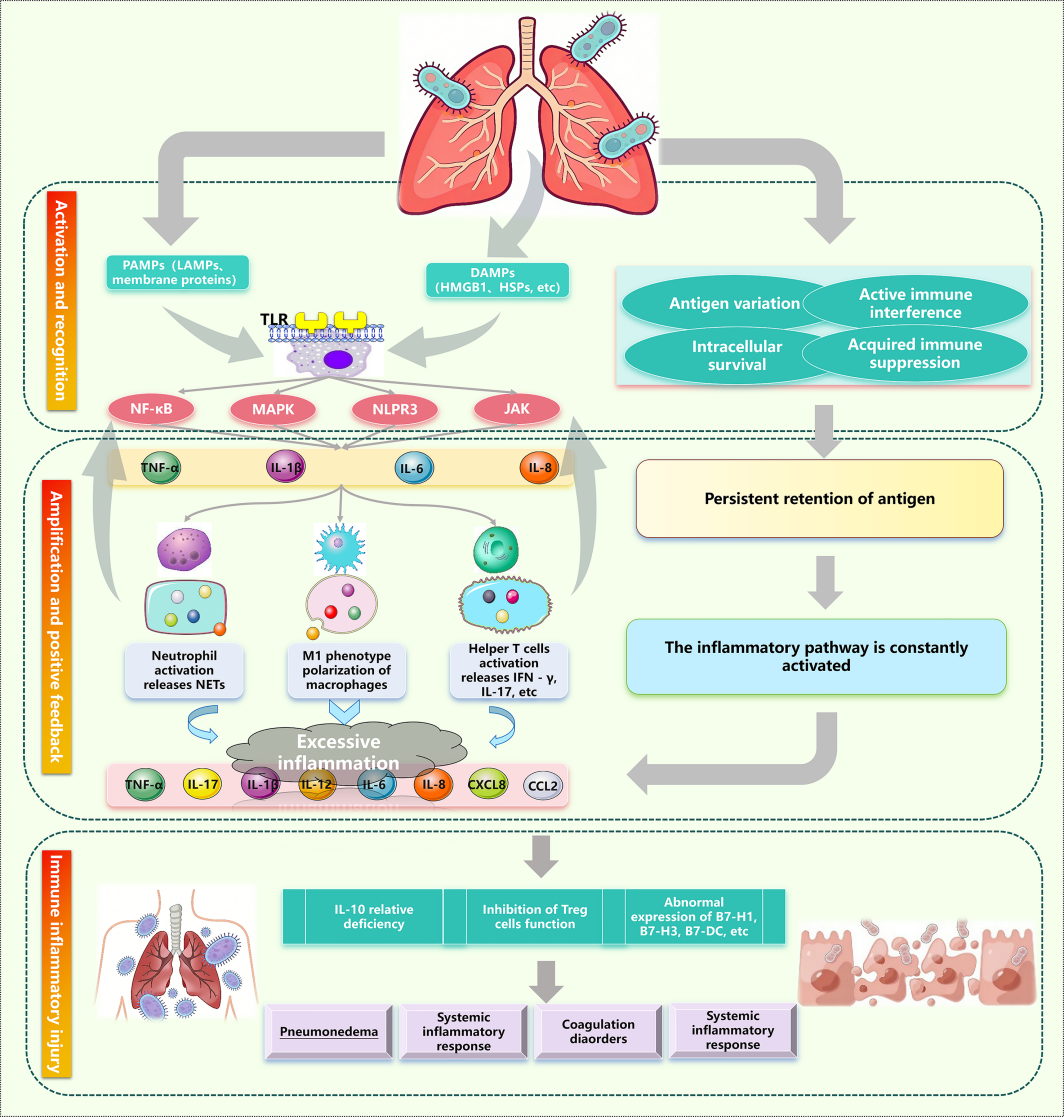
MP’s immune evasion strategies and excessive inflammation.

Immune evasion by MP is a critical trigger for initiating the inflammatory cascade. This cascade originates from the persistent retention of MP antigens: first, due to inadequate immune clearance, MP and its components serve as a persistent antigen reservoir, activating innate immune cells and inducing local malignant/excessive inflammation through inflammatory amplification pathways (98, 99). See **Figure 4**. Concurrently, host extracellular vesicles depend on TLR2-NF-κB/JNK signaling pathways to sustain inflammatory responses and intercellular immune regulation (100). Clinical evidence stems from persistently elevated levels of TNF-α and IL-8 in convalescent pediatric patients (101). Subsequently, ineffective immune clearance and sustained inflammatory drive trap the body in a “persistent response without resolution” state, ultimately causing structural alterations such as airway remodeling, bronchiectasis, and fibrosis (102). Under conditions where MP survives, immune escape can occur at any time, serving as the intermediate bridge in the pathological transition from excessive inflammation to chronic injury. The dual-edged role of autophagy in MP infection remains a major controversy. Whether autophagy promotes MP survival or enhances host clearance depends on the stage of infection, the infected cell type, and the integrity of autophagic flux; however, the specific mechanism remains unclear. The resolution of the dual effects of autophagy is still unclear. Within the tumor microenvironment, autophagy has been demonstrated to exhibit temporal duality (early suppression, late support) (103). The conditions governing this transition in MPP remain unclear. MP-induced autophagy typically exacerbates inflammatory responses, particularly in macrophages, and relies on TLR4 (104, 105). Conversely, enhanced autophagy in airway epithelial cells protects the airway epithelial barrier (106). This cell type-specificity of autophagy remains poorly understood in MPP. Future research should explore the effects of autophagy at different stages of MPP and its role in various cell types. Second, the mechanisms by which immune response heterogeneity determines clinical outcomes remain unclear. Particularly, the dynamic changes and context-dependent nature of Th1/Th2 responses. Most evidence supports an enhanced Th1-type T cell response during the acute phase of MPP infection (37); however, if the host is sensitized to allergens prior to infection, a robust and persistent Th2 response develops (60). This complex immune response pattern suggests that future immunomodulatory therapies should not focus solely on suppressing either Th1 or Th2 responses. Instead, they should be based on precise assessments of individual patient immune phenotypes (such as Th1/Th2/Th17 balance) and disease stages (acute hyperinflammatory phase or chronic immunopathological phase). At the level of immune recognition, MP’s regulation of TLR signaling is also debated. Some evidence supports “passive recognition” (98) while others argue for “active interference” (105). Finally, the role of Tregs remains controversial. Previous studies suggest that Tregs can suppress excessive inflammation and reduce tissue damage. However, evidence also indicates they may promote immune tolerance, leading to persistent MP infection (107). This contradiction is related to timing or metabolic context: during the acute phase, moderate Treg expansion suppresses excessive inflammation; whereas in the chronic phase, sustained or excessive Treg expansion inhibits effective antimicrobial immunity, resulting in delayed pathogen clearance (108). In tuberculosis, Tregs proliferate rapidly during early infection, but subsequent host responses selectively eliminate these highly suppressive Tregs, preventing them from limiting immunity in later stages (109). The changes Tregs undergo during different stages of MPP remain to be elucidated. The suppressive function of Tregs is highly dependent on CTLA-4; in CTLA-4-d eficient mice, Treg inhibitory activity is lost (110). This effect provides insights for studying the MP immune tolerance environment.

### 3.2 Dysregulation of inflammatory cytokines—the core of excessive immune inflammatory responses

Acute dysregulation of MPP typically occurs during the early phase of infection (1–2 weeks), characterized by excessive or dysregulated activation of the host innate immune system: (i) At the immune level, this primarily involves excessive cytokine release alongside overactivation of innate immune cells, initiating multifaceted proinflammatory cascades including NLRP. (ii) At the clinical biomarker level, besides acute phase manifestations (acute high fever, cough, dyspnea) and dramatic elevations in CRP, PCT, LDH, IL-6, PT, D-dimer, ferritin, etc. (111), Th17 cell activation has been identified as involved in acute MP clearance and excessive inflammatory responses (112). An increased proportion of CD4+ T cells and a significant decrease in CD8+ T cells are commonly observed in BALF. However, in children with severe MPP, T cell exhaustion in BALF can be observed (90). (iii) At the tissue level, changes are characterized by massive neutrophil and macrophage infiltration and fibrinous exudate in the alveolar spaces. This stage is not a linear, discrete phase but exhibits overlap and context-dependence. The intensity, duration, and outcome of this phase are determined by host-pathogen-environment interactions (113). In particular, children’s excessive susceptibility to inflammation in MPP is closely associated with their developing immune regulatory mechanisms.

#### 3.2.1 Pathogen-associated molecular patterns and damage-associated molecular patterns

MP carries multiple PAMPs, including LAMPs, membrane surface proteins, and nucleic acid components, which are continuously recognized by host pattern recognition receptors (114). In the context of immune evasion, PAMPs persistently linger in the respiratory tract, leading to abnormal and sustained activation of innate immune cells. Studies indicate that MP can persist and replicate in HEp-2 cells for at least six months (115); Liu et al. (53) found that MP-DNA can persist in the lower respiratory tract for up to four months. Additionally, the death of respiratory epithelial cells induced by MP infection releases large quantities of damage-associated molecular patterns (DAMPs), such as high mobility group box 1 (HMGB1), ATP, heat shock proteins (HSPs), and S100 family proteins, into the extracellular microenvironment, triggering proinflammatory responses (116). HMGB1 can bind to the receptor for advanced glycation end products (RAGE), triggering further release of cellular inflammatory mediators (117, 118). DAMPs also synergize with PAMPs to activate the NLRP3 inflammasome, continuously amplifying inflammatory signals and prolonging immune responses (119).

#### 3.2.2 Core effect cells and key inflammatory mediators

PAMPs promote macrophage polarization toward the M1 phenotype, inducing massive production of TNF-α, IL-6, IL-12, and chemokines, thereby forming a potent “pro-inflammatory mediator network” that establishes the material basis for “excessive inflammation” (100). Clinical studies have revealed that IFN-γ levels in BALF are significantly elevated in children with MPP, which can stimulate M1 macrophages via STAT1 to produce CXCL10. CXCL10 recruits Th1 cells to the inflammation site, inducing excessive release of pro-inflammatory factors (120). During the acute phase, MP infection also induces IL-23 production by alveolar macrophages. IL-17/IL-17F production is dependent on IL-23 and contributes to neutrophil recruitment (121). Peripheral blood mononuclear cells from MPP patients showed elevated Notch ligand DLL4 expression, positively correlated with IFN-γ and IL-17 levels, particularly in severe cases, indicating its role in amplifying Th1/Th17-mediated excessive immune responses (122). Recent studies indicate that MP-infected macrophages may undergo metabolic reprogramming (123), which provides the energy required for the sustained production of inflammatory cytokines. Compared to adults, neonatal alveolar macrophages are immature, and neonatal mouse alveolar macrophages retain high Nur77 expression, which negatively impacts macrophage inflammation and lung injury (124). Neutrophil extracellular traps (NETs) are also activated by MP, serving as key mediators of excessive inflammation (125). Mitochondrial DNA, a key structural component of NETs, can induce post-injury inflammatory responses and activate neutrophils. Activated neutrophils form NETs during inflammation and act as DAMPs to activate immune cells through TLR9 and cGAS-STING pathways, thereby establishing an inflammatory amplification cycle (126). Among these, excessive IL-17 release leads to sustained neutrophil recruitment, degranulation, and tissue damage, thereby exacerbating the inflammatory role of NETs in MPP (127). Clinical studies indicate that neutrophil and NET levels in PB correlate positively with CRP, LDH, D-dimer, and fever duration, and that these factors serve as risk factors for PB (128). The mycoplasma membrane fraction triggers neutrophil-mediated inflammatory responses by activating transcription element activator protein-1, nuclear factor-IL-6, MAPK, and NF-κB in BEAS-2B cells to increase IL-8 expression (129). Pulmonary microvascular endothelial cells are also activated, upregulating ICAM-1 and VCAM-1, the latter of which reprograms monocyte differentiation through NF-κB signaling, thereby mediating leukocyte endothelial cell adhesion (130). In summary, the excessive release of inflammatory mediators induced by MP infection in macrophages, neutrophils, and endothelial cells represents a central mechanism in the amplification of the inflammatory response.

#### 3.2.3 Abnormal amplification and positive feedback loops in cytokine signaling pathways

The JAK/STAT signaling pathway is strongly activated following MP infection. Validation in mouse models of MPP suggests that the CXCL12/CXCR4 axis activates both JAK-STAT and NF-κB pathways to exert synergistic proinflammatory effects, making it a key therapeutic target for MPP (131). Th1 cell-derived IFN-γ enhances macrophage CXCL10 production via the JAK-STAT1 pathway, thereby recruiting additional immune cells to the infection site, intensifing the Type I inflammatory response in MPP patients (120). MP also activates the STAT6-STAT3 signaling pathway, inducing the expression of the mucins MUC5AC and MUC5B, thereby downregulating FOXA2, leading to excessive airway mucus secretion, which exacerbates airway infections and is a major cause of mucus obstruction in various chronic airway diseases (132).

Persistent PAMPs and early inflammatory mediators in MP promote uncontrolled NF-κB activation, forming a difficult-to-interrupt inflammatory cycle (133). Research indicates that the MP MPN606 protein stimulates RAW264.7 cells to release nitric oxide (NO), induces M1-type macrophage activation, and activates the NF-κB and MAPK pathways, thereby promoting the secretion of proinflammatory factors (134). Its active lipoprotein component, the F0F1-ATPase, triggers excessive inflammatory responses by inducing NF-κB activation through TLR1, TLR2, and TLR6 (135). TLR expression varies across different host ages (136), increasing the risk of excessive inflammation in young hosts. MP also downregulates RECK expression by inducing Sp1 phosphorylation, modulating the NF-κB signaling cascade and increasing MMP-9 activity, thereby regulating inflammatory responses and promoting airway remodeling (137).

The vicious cycle of inflammation and oxidative stress also represents a significant pathway for MPP tissue damage. Clinical studies have revealed significantly elevated levels of malondialdehyde (MDA) and advanced oxidation protein products (AOPP) and reduced levels of superoxide dismutase (SOD) and glutathione peroxidase (GSH-PX) in BALF from children with MPP, forecasting that advanced oxidation protein products may serve as predictive biomarkers for disease severity in SMPP and RMPP (C-index = 0.960 (95% confidence interval 0.958–0.963) (138). MP’s MPN668 is a cysteine-based peroxidase whose gene encodes a protein with organic hydrogen peroxide reductase function (139). MP infection can induce mitochondrial dysfunction and activate NADPH oxidase (NOX) or directly suppress host catalase activity, leading to massive ROS production (140). This oxidative stress pathway further interacts with the NLRP3 inflammasome and NF-κB, exacerbating epithelial barrier disruption and immune cell infiltration, ultimately leading to chronic airway remodeling and fibrosis.

#### 3.2.4 Failure of immune homeostasis regulation mechanism

The failure of immune homeostasis regulation mechanisms further contributes to “excessive inflammation.” The compensatory insufficiency or functional defect in anti-inflammatory factors constitutes a key feature of immune homeostasis imbalance during the acute phase of MPP. IL-10 is a potent anti-inflammatory factor, yet it undergoes a dynamic evolution across different stages of MPP: During the acute inflammatory flare-up, serum IL-10 levels rise compensatorily in an attempt to suppress inflammation (54). However, both the intensity and duration of this response prove insufficient. Moreover, relative to the surge in TNF-α levels, IL-10 forms a “relatively deficient” level, rendering it incapable of counteracting the severe “excessive inflammation.” As the disease progresses, particularly in RMPP, this compensatory mechanism breaks down, manifested by the inability to maintain IL-10 levels or even a significant decrease, highlighting its anti-inflammatory deficiency (141). IL-10 and Treg cells synergistically exert immunosuppressive effects, but in MPP, immune homeostasis collapses. In the presence of IL-6 and TGF-β1, MP enhances IL-17A and IL-10 production in a concentration-dependent manner, which correlates with the development of extrapulmonary complications (99). Host heterogeneity manifests as the expression of extracellular adenosine in newborns, which suppresses Th1 cytokine production while enhancing Th2 and Th17-biased cytokine production compared to adults (136).

Treg cells in MPP exhibit increased numbers of activated cells but functional suppression (142). A Chinese study reported that children with MPP exhibited significantly elevated peripheral blood levels of CD4+ CD25+ Tregs; nonetheless, these increases failed to effectively control inflammation (143, 144). Although CD4^+^ CD25^+^ FoxP3^+^ T cells can produce IL-10 and TGF-β to exert anti-inflammatory effects, they can also promote IFN-γ and IL-17 responses (145). MP infection causes a significant decrease in serum TGF-β1 levels in children, and weakens the ability to induce and activate CD4+CD25+Treg, leading to a decrease in immune suppression function and an increase in immune response, resulting in thrombocytopenia (146).

Immune checkpoint molecules (PD-L1, B7-H3, and B7-DC) are key regulators of T cell activation, maintaining self-tolerance and preventing excessive inflammation. In severe MPP cases, functionally exhausted T cells frequently interact with macrophages expressing high levels of PD-L1, synergistically suppressing the host’s ability to eliminate MP through the PD-1/PD-L1 “braking” signal (147). Similarly, B7-DC binding to PD-1 inhibits TCR-mediated T cell proliferation and cytokine production, thereby reducing T cell activity. MP induces high B7-DC expression, which co-stimulates CD4^+^ T cell responses via RGMb, promoting Th1 polarization and enhancing IFN-γ secretion (148, 149). B7-H3 positively correlates with TNF-α and IL-17 levels in MPP (150). The abnormal expression of checkpoint molecules disrupts the balance between immune activation and suppression, prolongs acute inflammation, and contributes to chronic sequelae such as post-infectious cough and asthma-like symptoms (151).

#### 3.2.5 Controversial issues targeting “excessive inflammation”

A current debate concerns the origin of the inflammatory dysregulation. Is it primarily pathogen-driven or host-dominated? The pathogen-centric theory emphasizes the direct roles of specific virulence factors, CARDS Tx, and adhesion mechanisms (98). The host-dominant theory posits that the host’s genetic background and immune status are decisive factors in whether “inflammatory dysregulation” occurs (152). As a “relatively weak pathogen,” MP acts to ignite a pre-existing immunological “powder keg.” Genome-wide association studies have linked specific HLA genotypes and polymorphisms in innate immune signaling molecules (TLRs and IL-1R) to increased susceptibility to RMPP (153). Post-COVID-19 era data from China reveal that near-100% macrolide resistance and increased prevalence of the 4-5-7–2 genotype (enriched in virulence and metabolism-related genes), driving the resurgence of MP after the epidemic (154). Animal studies indicate that BALB/c mice exhibit significantly higher levels of pulmonary inflammatory cell infiltration, BAL cell counts, and proinflammatory cytokine release (TNF-α, KC, IFN-γ) compared to C57BL/6 mice (152). This explains why only some children develop a malignant inflammatory response under identical infection conditions. On the other hand, within the highly complex inflammatory regulatory network of MPP, a universal, upstream “master switch” pathway remains elusive. Crucially, pathways activated in laboratory settings have yet to be translated into clinically identifiable and actionable “immune phenotypes.” While animal models offer valuable insights, direct evidence from clinical samples remains limited. Circulating proinflammatory factor levels merely indicate the presence of inflammation without elucidating dominant signaling pathways, thus failing to guide targeted immunosuppressive interventions. Conducting multi-omics integration studies and immune cell profiling analyses based on clinical cohorts to establish “host response phenotypes” centered on specific signaling nodes is the cornerstone for constructing future precision immunotherapy systems. The effects of immune checkpoints also exhibit dual-mode conversion: in chronic infections, cytotoxic CD8+ T cells become exhausted and highly express PD-1, preventing pathogen clearance (155, 156). In acute viral infections, activated PD-1-positive effector cells do not become exhausted, and activated cytotoxic CD8+ T cells moderately upregulate PD-1 to exert potent antiviral immune responses (157), but whether this effect is similarly replicated in MPP remains unreported. Furthermore, the relationship between “excessive inflammation” and macrolide resistance remains unclear. Clinical observations suggest that infections caused by resistant MPP are more likely to cause severe disease (158); however, the underlying mechanism remains to be elucidated.

### 3.3 Chronic immune impairment—from persistent inflammation to structural remodeling

**Figure 5:**
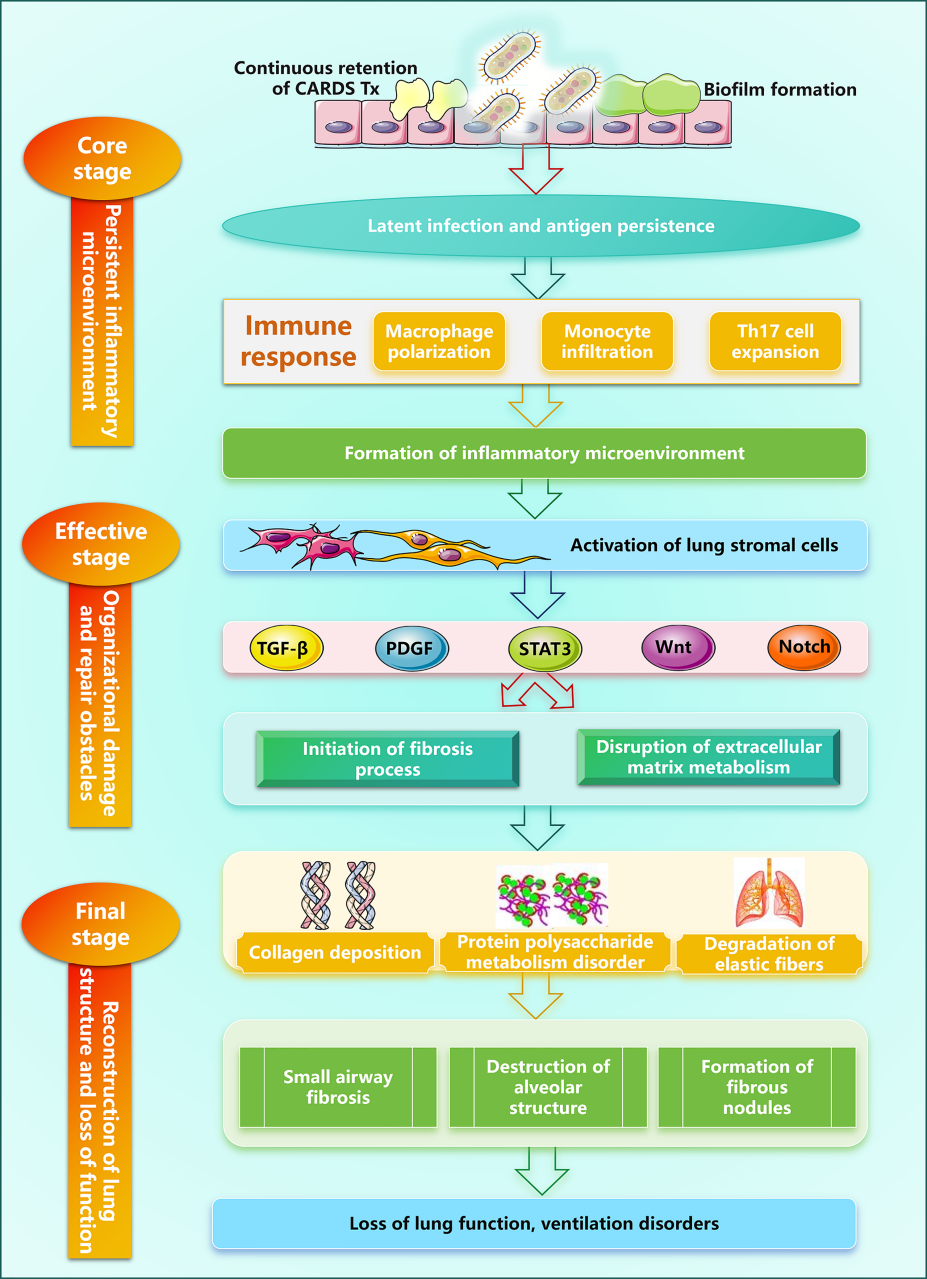
Schematic of MPP-induced chronic injury.

Following a complex network of immune evasion and excessive inflammation (**Figure 4**), MP infection ultimately leads to pulmonary structural remodeling and functional loss (**Figure 5**). This stage typically occurs 3–6 months or even longer after acute infection, but the key mechanism (NLPR3 overexpression promoting pulmonary fibrosis) may already be initiated during the acute phase or immune escape phase (159). The diagnosis of MPP chronic injury also requires multidimensional criteria: (i) At the immune threshold level, interactions between MP and airway epithelium generate a TGF-β1-dominant chronic immune microenvironment leading to excessive ECM deposition (160). (ii) Clinically and serologically, pulmonary function tests reveal persistent small airway obstruction or restrictive ventilatory impairment, while HRCT demonstrates irreversible mosaic perfusion, bronchial wall thickening, bronchiectasis, or early fibrotic reticular patterns (161). Abnormally elevated levels of salivary glycoprotein antigen-6 (KL-6), a gold standard biomarker for type II alveolar epithelial cell injury/regeneration, and upregulation of mucin MUC5B constitute risk factors for persistent pulmonary sequelae (162). (iii) Tissue-level alterations, including characteristic pathological changes of BO, interstitial fibrosis, and abnormal lymphoid follicular hyperplasia (163). At this stage, the contribution of immune escape to chronic immune activation, fibrosis, and airway remodeling cannot be overlooked.

#### 3.3.1 Formation of the persistent inflammatory microenvironment

The absence of a cell wall and unique immune evasion mechanisms enable MP to achieve long-term colonization and persistent infection within the host respiratory tract. In particular, CARDS Tx can persist in lung tissue for up to 18 months, sustaining a chronic inflammatory/chronic immune activation state locally (164). Additionally, MP can form biofilm-like structures that enhance antibiotic resistance and immune evasion capabilities, leading to low-level pathogen replication and persistent antigen exposure, while promoting chronic inflammatory cell infiltration and abnormal polarization of immune cells (165). Animal studies indicate that MP-induced chronic infection in BALB/c mice can persist for at least 18 months, with pulmonary histology revealing perivascular and peribronchial mononuclear cell infiltration, sustained positive IgG titers, elevated IL-4 concentrations, and airway hyperresponsiveness and functional airway obstruction in mice (166), this chronic respiratory infection likely stems from MP’s intracellular survival strategy. Against this backdrop, multiple immune cell populations abnormally accumulate in lung tissue and undergo functional polarization. Macrophages undergo pronounced M2 polarization after MP infection, which upregulates arginase-1 (Arg-1) expression and inhibits T cell function, while promoting the release of profibrotic factors such as TGF-β and platelet-derived growth factor (PDGF), mediating matrix deposition and tissue remodeling (167). Chronic infection established by low-dose MP enhances IL-4 expression in CD4+ T cells and eotaxin-2 expression in BALF of allergic mice, promoting pulmonary eosinophil infiltration to exacerbate Th2 responses (168). Within adaptive immunity, Th17 cells and follicular helper T cells expand markedly in MPP. The former secrete IL-17A and IL-17F, recruiting neutrophils and activating fibroblasts (169); the latter promote abnormal B-cell activation and plasma cell differentiation via IL-21 and ICOS signaling, leading to excessive local antibody production and hypergammaglobulinemia (170).

Simultaneously, the release of chronic inflammatory mediators continuously activates pulmonary stromal cells and sustains the inflammatory state. IL-17 synergizes with TNF-α to enhance inflammatory effects (171). CCL2 persistently recruits monocytes, which differentiate into macrophages and participate in the inflammatory response (172). Ultimately, sustained antigen exposure, in concert with inflammatory mediators, acts on structural cells within the lung, inducing EMT-like changes in airway epithelial cells, transforming pulmonary fibroblasts, and upregulating adhesion molecules in vascular endothelial cells, thereby leading to excessive extracellular matrix deposition and tissue structural remodeling (173).

#### 3.3.2 Initiation of fibrosis and ECM metabolism

MP can upregulate TGF-β1 expression (174). TGF-β1 induces the expression of ECM proteins through the classical Smad signaling pathway and non-Smad pathways, regulates ECM degradation by modulating MMPs and their tissue inhibitors, and promotes the production of connective tissue growth factor (CTGF), leading to ECM accumulation and fibrosis (175, 176). The persistent antigen repertoire established by immune evasion leads to sustained chronic immune activation (164, 165) which further exacerbates fibroblast activation and ECM deposition in a TGF-β-dominant pro-fibrotic microenvironment, ultimately triggering tissue remodeling (175, 176). It has been discovered that chronic infections established through immune evasion can regulate the HSF1/HSP70 axis via TLR2 signaling to mediate pulmonary fibrosis (177, 178). miRNAs, lncRNAs, and circRNAs may mediate MP-induced pulmonary fibrosis, but the specific mechanisms remain to be elucidated (179). Chronic MP infection induces pathological vascular remodeling and microenvironmental hypoxia in the lungs, which can upregulate expression of multiple pro-fibrotic genes (such as VEGF, PDGF, and TGF-β) (180) or promote vascular remodeling and fibrosis by regulating expression of angiopoietin-1, IL-6, MMP2, collagen I, and collagen III through the calpain-1-HIF-1α axis (181). Despite the discovery of MP’s specific role in promoting fibrogenic factors, direct evidence for MPP-induced pulmonary fibrosis remains limited. Future research should establish chronic infection models to elucidate the precise mechanisms by which MP drives fibrosis and the unique role of immune evasion.

MP infection can also affect ECM metabolism. During the chronic phase of MPP, TGF-β1 activates the Smad signaling pathway to upregulate collagen genes such as COL1A1 and COL3A1, thereby driving excessive synthesis of type I and III collagens (182, 183). Recurrent MP infection may lead to increased levels of type I collagen and type I/III collagen ratio, contributing to pulmonary arteriolar remodeling and pulmonary interstitial fibrosis (184). Elastic fibers are key ECM components that maintain lung tissue elasticity and reversible deformation. However, during the chronic phase of MPP, neutrophils and macrophages secrete large amounts of MMP-9 and MMP-12 to enhance elastin degradation, disrupting the elastic fiber network in alveolar and airway walls (185). Elastin degradation products can also serve as DAMPs recognized by the TLR4 signaling pathway, further amplifying the inflammatory response and promoting myofibroblast differentiation (186), ultimately accelerating the decline in lung function.

#### 3.3.3 Organ-specific structural remodeling

Following MP infection, the small airways—particularly bronchioles—become primary targets for chronic inflammation and fibrotic remodeling. During chronic recovery phase, large amounts of activated fibroblasts and myofibroblasts accumulate in the airway wall and excessively deposit ECM, leading to diffuse thickening of the airway wall and narrowing of the lumen. In more severe cases, characteristic pathological changes—mosaic-like patterns—may develop in the bronchioles, leading to irreversible airflow limitation (187). Concurrently, alveolar epithelial cells, especially type I cells, undergo extensive shedding and necrosis, while type II epithelial cells fail to proliferate and restore a functional barrier, leading to alveolar septal rupture, alveolar cavity fusion, and even cystic changes or emphysematous alterations (188). The bronchial walls also undergo dysplasia and remodeling due to chronic inflammatory stimulation. During the repair of pseudostratified ciliated columnar epithelium, it is replaced by stratified squamous epithelium, but loses its ciliary clearance function. This further weakens the airway’s defense capabilities, creating conditions for pathogen re-colonization and persistent inflammation (189). Additionally, cup cell hyperplasia leads to excessive mucus secretion, which together with ciliary dysfunction contributes to mucus plug formation. This plays a key role in RMPP complicated with PB (190). Importantly, children have narrower airways and underdeveloped elastic fibers in their lung tissue. Although their tissue possesses greater repair and regenerative capacity, a dysregulated inflammatory environment may redirect this active repair process toward pathological fibrosis (191). Structural remodeling may develop on the basis of chronic infection established by MP immune evasion, but evidence remains scarce regarding how immune evasion specifically influences pulmonary structural remodeling. The Th2 response alone cannot fully account for this complex process.

#### 3.3.4 Controversial issues for chronic injuries

The key transition points in the pathophysiological conversion of chronic immune injury in MPP remain highly controversial. Some scholars propose that chronic fibrosis represents abnormal tissue repair following acute excessive inflammation -induced damage. Others suggest that MP infection may independently initiate fibrosis through epigenetic modifications (for example, DNA methylation) or metabolic reprogramming (192, 193), allowing progression even after acute inflammation resolves. This debate directly influences therapeutic strategy selection: If it is a continuation of the acute phase, early anti-inflammatory intervention becomes critical; if an independent mechanism exists, should new therapies targeting fibrosis-specific pathways be developed? However, the practical challenge is that the vast majority of studies remain confined to the acute phase or cross-sectional designs, with a severe lack of continuous, dynamic human data spanning from the acute infection phase to the chronic sequelae phase. It remains unknown whether fibrosis-associated molecules identified in animal models are equally critical or detectable in pediatric patients. No data exist on the optimal timing or duration of intervention—whether anti-inflammatory or anti-fibrotic—to achieve the best benefit-risk ratio. Furthermore, there are no clinical clues regarding the direct applicability of adult anti-fibrotic drugs in children (194). It must be acknowledged that a substantial evidence gap currently exists for advancing any specific intervention. The most pressing gaps involve the lack of longitudinal human data revealing the chronic disease dynamics, validated biomarkers for clinical subtyping, and safe intervention timing and protocols for children. Future research must prioritize filling these gaps to enable precise prevention and treatment of chronic sequelae of MPP.

In summary, immune evasion, dysregulated inflammation/excessive inflammation, and chronic injury collectively constitute the overall landscape of immune imbalance throughout the entire course of MPP. The relevant mechanisms are summarized in **Table 2**.

**Table 2:** Mechanism of MPP immune dysregulation.

Number	Classification of mechanisms	Research design	Details	References
1	Immune Evasion	Vitro Study	P1 high-frequency variants: RecAMp and RecAMg homologous recombination.	(59)
2		Vivo Study	CARDS Tx immune interference: autoimmune reaction.	(60)
3		Vitro Studies	LAMPs domain variation: decreased NF-κB activation, failure of TLR2 to recognize FSL-1.	(61, 62)
4		Vitro Studies	Physical camouflage: interaction of CPS with the DC-SIGN pathway.	(63, 64)
5		Clinical Trials, Vivo Studies	Molecular mimicry:Glyceraldehyde-3-phosphate dehydrogenase, Gal-C-like glycolipid structure cross-reacts with the host.	(65, 66)
6		Vitro Study	Factor H evades the complement system.	(67)
7		Vitro Study	Oxidative stress versus antioxidant.	(68)
9		Vitro Studies	Apoptosis and autophagy: inhibition of host cell apoptosis, inhibition of autophagosome-lysosome fusion.	(69, 70)
9		Vitro Study	Intracellular survival: internalization of CARDS Tx, host acidic pH environment and vacuolar ATPase enhance ice nucleation activity.	(36)
10		Vitro Studies	Intracellular survival: IbpM binds to host immunoglobulin, Mpn491 evades neutrophil killing.	(79, 80)
11		Vitro Studies	Dendritic cell dysfunction: HMGB-1 and MALP-2 induce DC maturation, CPS inhibits DC maturation.	(85–87)
12		Clinical Trial	T cell dysfunction: CD3^+^ and CD4^+^ T cells decrease, CD8^+^ T cells increase.	(89)
13	Inflammatory Dysregulation	Clinical Trial	PAMPs long-term retention: MP-DNA can persist in the lower respiratory tract for up to 4 months.	(53)
14		Clinical Trials, *In Vitro* Studies	LAMPs pro-inflammatory response: HMGB1 binds to RAGE/TLR.	(117, 118)
15		Clinical Trial	Macrophage M1 phenotype polarization: upregulation of IFN-γ and CXCL10 expression, Th1-type inflammatory response.	(120)
16		Vivo Study	Macrophage: secretes IL-23, promoting the recruitment of neutrophils	(121)
17		Clinical Trial	Monocytes: Upregulate DLL4 to enhance Th1/Th17-mediated immune responses	(122)
18		Clinical Trials	NETs amplify inflammatory cycle: neutrophil and NET levels are upregulated and positively correlate with CRP, LDH, D-dimer, and fever duration.	(127, 128)
19		*In Vitro* Studie	Neutrophil inflammatory response: MMF activating protein-1, MAPK, NF-κB increase IL-8 expression.	(129)
20		Clinical Trials, *In Vitro* Studies	JAK/STAT signaling pathway activation: CXCL12/CXCR4 axis mediates RMPP occurrence, STAT induces MUC5AC and MUC5B causing airway mucus hypersecretion.	(131, 132)
21		*In Vitro* Studies	NF-κB dysregulation: MPN606 induces macrophage M1-type activation, F0F1-ATPase activates TLR1, TLR2, and TLR6, MP downregulates RECK expression.	(134–137)
22		Clinical Trial	Oxidative stress: upregulation of MDA and AOPP, downregulation of SOD and GSH-PX.	(138)
23		Clinical Trials	Immune homeostasis imbalance: compensatory upregulation of IL-10 in the acute phase and deficiency in the severe phase.	(54, 141)
24		Clinical Trials	Treg cell function inhibition: upregulation of CD4+CD25+ Treg and Th17/Treg ratio	(142–144)
25		Clinical Trials	Immune checkpoint molecule abnormality: high expression of PD-L1, B7-DC, and B7-H3.	(147, 148, 150)
26	Chronic Injury	Vivo Studies	Antigen persistent infection: MP-induced chronic infection state in mice can last for up to 18 months.	(166)
27		Clinical Trial	Promotes fibrosis initiation: TGF-β upregulation	(174)
28		Vitro Studie	Reverse evidence: MP infection of MRC-5 cells stimulates miR-145 expression, negatively regulating the TGF-β/Smad pro-fibrotic pathway.	(175)
29		Vivo Studies	Airway collagen deposition: upregulated by MP infection.	(182)
30		Vivo Studies	Structural remodeling: MP infection of Wistar rats induces pulmonary arteriole remodeling, pulmonary hypertension, and pulmonary interstitial fibrosis.	(184)
31		Clinical Trial	BO serious complications: wheezing, mosaic sign, central bronchiectasis, and emphysema.	(187)

## 4 Current status of MPP clinical translation

### 4.1 Immunobiological markers and disease severity/clinical phenotype assessment

The heterogeneity of MPP clinical manifestations reflects variations in host immune responses. Studies indicate that although MP DNA load shows no correlation with MP genotyping, it is significantly associated with clinical phenotypes (195): the high-load group exhibited longer hospitalization duration, higher peak fever temperatures, and elevated inflammatory markers (CRP, PCT, AST), particularly IL-6, which demonstrated a linear correlation with MP DNA load. An intrinsic relationship between IL-6/IL-17A and four clinical phenotypes (segmental pulmonary lesions, segmental pulmonary lesions with necrosis, diffuse bronchiolitis, and mild lesions) has also been identified (196), which remains a consequence of excessive host immune responses. Another review examined the differential expression of lymphocyte subsets, including CD3+, CD4+, and CD4+/CD8+ levels, between the acute and convalescent phases, as well as between severe and mild MPP cases (197). Predictive models based on CD3-CD19+% and monocyte counts may play a crucial role in the early diagnosis of severe MPP, particularly in children aged ≤5 years (198). Proteomics revealed that CD209, CHM, PBRM1, and SCAMP1—proteins involved in immune responses and inflammatory signaling—are the most influential predictors of MPP severity (199). Although these potential immune biomarkers have been identified, further research is needed to determine whether these clinically actionable markers can be used to immunophenotype MPP and distinguish the severity of its clinical phenotypes—specifically, “T cell exhaustion” in severe patients versus “neutrophil activation” in mild patients (148). Prior to this, the following gaps must be filled to enable immune phenotype-guided clinical decision-making: (i) Using standardized testing protocols to longitudinally track the dynamic changes of these biomarkers, establishing their stable association with disease progression; (ii) Validating the causal relationship between immune status and inflammatory tissue damage through preclinical models and targeted intervention studies; (iii) Excluding clinical confounding factors to confirm the predictive value of target immune biomarkers for disease severity; and (iv) Demonstrating that interventions based on such immune phenotyping can improve prognosis.

### 4.2 Anti-inflammatory treatment for immune-mediated inflammation and the controversy surrounding glucocorticoid use

The pathological features of immune-mediated hyperinflammation underscore the necessity of anti-inflammatory therapeutic strategies. The combination therapy of macrolide antibiotics with glucocorticoids has demonstrated potential advantages. Wu et al. (200) demonstrated that the combination of budesonide and azithromycin significantly alleviates clinical manifestations of pediatric MPP and reduces serum IL-6 levels, reflecting the combined effects of immunomodulation and anti-inflammatory mechanisms. The combination of Pulmicort and azithromycin improves IgG, IgA, and IgM levels in children with recurrent respiratory infections caused by MP (201). This suggests that glucocorticoids may enhance the therapeutic efficacy of macrolide antibiotics (202). Wei et al. (203) investigated the relationship between inflammatory markers and glucocorticoid dosage at admission, finding that the high-dose group (≥10 mg/kg/d) exhibited significantly higher levels of white blood cells, C-reactive protein, procalcitonin, lactate dehydrogenase (LDH), alanine aminotransferase, aspartate aminotransferase, ferritin, erythrocyte sedimentation rate, and D-dimer levels were significantly higher than those in the low-dose group (≤2 mg/kg/d) and medium-dose group (2–10 mg/kg/d), and patients in the high-dose group also exhibited more severe imaging findings, longer hospital stays, and higher rates of hypoxia (*P* < 0.05). This finding is crucial for selecting glucocorticoid dosing based on clinical phenotype. Glucocorticoids can rapidly improve clinical symptoms and chest X-ray findings through their anti-inflammatory and immunomodulatory effects (204, 205). However, their use remains controversial, primarily concerning how to achieve a precise balance between risks and benefits: First, timing of initiation—should early intervention be based on high clinical risk factors, or should one wait for definitive inflammatory biomarkers (IL-6, CRP, etc.)? Second, patient selection: Should all patients with severe radiographic findings receive treatment? Do those with mild disease but markedly elevated inflammatory markers benefit? Furthermore, concerns persist regarding secondary infections, blood glucose disorders, and potential masking of signs of infection.

For potential immune marker stratification, cell-mediated inflammatory factor antagonists may represent a potential therapeutic strategy. Preliminary findings indicate that monoclonal antibodies targeting IL-6 receptors and IL-1 receptors demonstrate initial efficacy in inflammatory diseases (154, 206). Case reports suggest that they alleviate systemic inflammation and lung injury in severe MPP. Polyclonal antibodies targeting the P116–661 protein effectively inhibit adhesion between MP and A549 cells, reduce the secretion of inflammatory mediators such as IL-6 and TNF-α, and improve pulmonary pathology (207). This provides novel experimental evidence for anti-inflammatory immunotherapy against MP infection, though it remains confined to preclinical research. The potential time window for using these biological inhibitors, along with safety concerns, still requires long-term, large-scale clinical trials for further validation. Before implementing anti-inflammatory immunotherapy, it is imperative to resolve evidence gaps affecting clinical translation, including dominant target selection, immune phenotype stratification, optimal timing of intervention, and assessment of efficacy and safety. There is an urgent need to establish early warning models based on integrated clinical-immunological biomarkers to guide the precise initiation and dose stratification of glucocorticoid therapy. Concurrently, for refractory cases unresponsive to corticosteroids, translational pathways for targeted cytokine therapies and novel immunomodulatory strategies should be explored. This will enable the construction of a tiered precision treatment framework spanning from assessment to intervention.

### 4.3 Clinical management insights for extrapulmonary complications

Immune-driven mechanisms, autoimmunity, immune complexes, and non-specific antibodies produced by B lymphocytes are key factors causing extrapulmonary multisystem immune impairment (such as glomerulonephritis and hemolytic anemia) (11). This immunopathological feature informs the clinical management logic for extrapulmonary complications of MP: shifting from purely antimicrobial therapy to a comprehensive strategy centered on “infection control as the foundation and immune modulation as the core.” Chen et al. (208) reported a case of MP infection complicated by severe neutropenia, thrombocytopenia, and hepatitis. These extrapulmonary manifestations were associated with autoantibodies and resolved following steroid therapy. MP infection may also trigger allergic purpura, which arises from immune complexes formed by abnormally glycosylated circulating IgA and IgG antibodies, which frequently leads to renal involvement (30–50% of pediatric cases), requiring treatment with glucocorticoids or immunosuppressive agents (209). Despite awareness of the risks associated with extrapulmonary complications, the current reality is that there remains a lack of clinical consensus on the management of MP-related extrapulmonary complications, particularly concerning related immunotherapies. There remains a need to systematically elucidate the immune pathogenesis of extrapulmonary complications in prospective cohort studies, identify specific early diagnostic biomarkers, evaluate the efficacy and safety of different immunomodulators in pediatric patients with specific mechanistic phenotypes, and establish long-term follow-up data to clarify the impact of various immune interventions on patient long-term outcomes.

### 4.4 Therapeutic challenges and novel immunological intervention strategies in the context of macrolide resistance

Given the current clinical reality of persistently high macrolide resistance rates (210), the treatment paradigm for MRMP has undergone a fundamental shift. The core therapeutic challenge is no longer merely “antibiotic failure, “ but rather the long-term colonization of MRMP due to ineffective anti-MRMP therapy (211, 212). Timely replacement with antibiotics that are sensitive to MRMP (tetracyclines and quinolones) can effectively shorten the duration of fever and the disease course (213). However, the excessive immune response caused by long-term MRMP colonization may alter clinical outcomes or increase the risk of disease severity and extrapulmonary complications (214). Therefore, current treatment strategies emphasize early identification of resistance risk (lack of response to 3 days of macrolide therapy should be considered indicative of MRMP) (215) and rapid initiation of effective antimicrobial therapy. A dual-track comprehensive management approach combining “antimicrobial intensification” (antibiotic escalation) with “immunomodulation” (glucocorticoids or gamma globulin) represents an effective therapeutic strategy for the clinical management of MRMP (215). A retrospective analysis compared the efficacy and safety of oral doxycycline, oral minocycline, oral doxycycline combined with intravenous corticosteroids, and oral minocycline combined with intravenous corticosteroids in treating severe MRMP pneumonia. Results showed that, regardless of corticosteroid combination, the doxycycline group achieved higher fever resolution rates at 24 and 48 hours than the minocycline group, and that the combination of newer-generation tetracyclines with corticosteroids significantly improved clinical symptoms, accelerated clinical and chest radiographic recovery, and prevented disease progression and complications (216). However, there remains a lack of multicenter, large-sample, evidence-based medical support for the combination of tetracycline and glucocorticoids in the treatment of refractory MRMP pneumonia.

Another noteworthy concern is that the intersection of drug resistance and immune evasion may exacerbate treatment challenges. Studies indicate that P1–2 genotype strains not only acquire macrolide resistance (for example, A2063G/A2064G mutations) more readily but also exhibit enhanced transmissibility (217). Whether these resistant strains also possess enhanced immune evasion capacity remains to be determined. Macrolide antibiotics themselves possess immunomodulatory effects (218). For instance, the immunostimulatory and epithelial cell-stimulating actions of azithromycin involve interactions between phospholipids and Erk1/2, as well as NF-κB regulation. Its delayed inhibitory effect on cellular function and high accumulation in lysosomes are frequently accompanied by disruption of protein and intracellular lipid transport, modulation of surface receptor expression, altered macrophage phenotype, and impaired autophagy (213). Long-term or widespread use may inadvertently alter the host immune microenvironment and even select for MP strains with superior immune escape traits, thereby complicating clinical efficacy assessments and mechanistic studies. Researchers have also identified associations between macrolide antibiotic resistance and high pathogen burden with exacerbated airway inflammation and immune dysregulation in MPP children (219). Therefore, attention should also be given to the relationship between the timing of antibiotic use for MRMP pneumonia and its immunomodulatory effects.

More precise novel immunomodulatory strategies represent the cutting edge of future clinical translation. However, some novel immunosuppressants remain in animal studies or in case reports. For instance, the JAK inhibitor tofacitinib downregulates CXCR4 expression on CD4+ T cells in MPP-infected mouse lungs, thereby reducing inflammatory responses (131). The chimeric recombinant protein HP14/30 reduces the average adhesion force of MP to HeLa cells to 6%, suggesting that optimized immunizations may further prevent MP host colonization (220). The lncRNA NNT-AS1 promotes MP-induced inflammatory damage in A549 cells via the miR-410-3p/TMEM14A/Wnt/β-catenin signaling pathway (221). It approaches targeting lncRNA expression regulation, including antisense oligonucleotides (ASOs), small interfering RNA (siRNA), and lncRNA delivery technologies such as nanoparticles or microvesicles, showing promising potential in MPP therapy. Future efforts should focus on targeting immune regulation in MPP, establishing MRMP infection models, and identifying reliable immune biomarkers to accelerate the development of novel immunomodulators.

### 4.5 Prediction of chronic lung injury risk and long-term health management based on immunological prognosis

Chronic lung injury resulting from MPP suggests that its clinical translation should extend to more profound “chronic risk prediction and long-term health management.” Xu et al. (222) established that lobar consolidation, diffuse bronchiolitis, superimposed infection, atopic disease, bronchial mucus plugging, CRP, mechanical ventilation, and fever duration are prominent independent risk factors for BO development following MP infection. Wu et al. (223) developed an integrated naming model demonstrating that age, APACHE II score, sputum color, mucosal edema, CT score, and PCT levels are crucial for early and accurate prediction of the risk of RMPP in children and limiting the progression of sequelae. However, most of the above studies are based on risk model predictions derived from acute-phase clinical phenotypes, and there remains a lack of chronic risk model predictions targeting immune biomarkers.

Based on precise prediction, the ultimate goal of translational research is to establish personalized long-term management plans and conduct regular assessments of immune function. This necessitates developing structured follow-up protocols for high-risk pediatric patients, including dynamic monitoring of lung function, airway inflammation, and key serum immune biomarkers. Furthermore, targeted secondary prevention or immunomodulatory interventions should be explored based on immune phenotypes, thereby achieving precision management through a “risk stratification-immune-guided intervention-dynamic immune monitoring-chronic injury prevention” approach. However, the synergy and contradictions within treatment strategies remain the core point of contention. Although anti-fibrotic drugs (such as pirfenidone) possess immunomodulatory potential, their role in chronic MPP injury remains unclear; targeting chemokines like CCL2 can block fibrosis (224) but may impair normal repair. Immune reconstitution therapies (e.g., low-dose IL-2 expansion of Tregs) (225) also carry risks of dysregulation and reinfection. Deeper challenges lie in two aspects: first, the reversibility of fibrosis remains uncertain, making the intervention “time window” difficult to define; second, efficacy assessment tools are inadequate, as current CT and pulmonary function tests struggle to reflect fibrosis activity and immune microenvironment changes early and sensitively. This hinders the clinical evaluation of novel therapies. The breakthrough lies in leveraging multi-omics technologies to identify biomarkers that can precisely and dynamically assess disease activity and treatment response. This would clarify optimal intervention timing, enabling a genuine shift from symptomatic treatment to disease-modifying interventions.

All in all, many proposed immune-guided interventions remain hypothesis-generating in pediatric MPP. Key gaps include lack of longitudinal human datasets, validated phenotype biomarkers, optimal timing/duration of interventions, and pediatric safety/risk–benefit evidence, including uncertainty about applying adult antifibrotic drugs in children.

## 5 Conclusions

The immune response landscape throughout the entire course of MPP is characterized by an overlapping network of immune dysregulation, immune evasion, and chronic injury. Leveraging large-scale randomized controlled trials, dynamic observational studies, and artificial intelligence technologies to predict and broadly distinguish the three pathological stages of MPP will facilitate clinical management and advance disease research. Age-related immune differences warrant attention for their impact on the three stages. Key controversies include the dual-edged role of autophagy (survival *vs.* clearance), the dynamic and context-dependent evolution of Th1/Th2 responses, the origins of inflammatory dysregulation (pathogen-centric *vs.* host-dominant theories), and the pathological mechanisms at transition points in chronic injury (persistence of the acute phase *vs.* independent progression). Key areas for clinical translation remain: integrating immune biomarkers with clinical phenotype assessment; combined application of anti-inflammatory therapy and immunomodulation; establishing consensus for managing extrapulmonary complications; addressing treatment challenges of “antimicrobial intensification” versus “immunomodulation” in drug-resistant settings; advancing novel targeted immune intervention strategies; and developing chronic injury risk prediction models alongside long-term management strategies. In the future, it is still necessary to reveal the age-related immune profile characteristics of MPP and address the differences in evidence such as immune immaturity and immune escape, immune subtype stratification, dominant target selection, and longitudinal human data of chronic dynamic processes.
